# Point-of-Care Ultrasound Diagnosis of Retrobulbar Hematoma: A Sight-Threatening Emergency

**DOI:** 10.1016/j.acepjo.2026.100527

**Published:** 2026-10-19

**Authors:** Prateek Khurana, Vivian Papas, Allison Cohen

**Affiliations:** Department of Emergency Medicine, Northwell Health Manhasset, New York, USA

**Keywords:** Ultrasound, Ocular trauma, Vision Loss

## 1 Case Presentation

A 93-year-old female with a past medical history of dementia and hypertension, not taking anticoagulation treatment, presented to the emergency department with a chief complaint of head injury after falling down a flight of stairs. On arrival, she was hemodynamically stable with an intact primary survey. On secondary examination, the patient had left frontal ecchymoses, a minimally reactive left pupil, and difficulty with left extra ocular movements. The patient denied any vision changes or ocular pain. Given the suspicion of orbital trauma, a computed tomography (CT) of the head and maxillofacial bones was ordered, and a point-of-care ultrasound (POCUS) was concurrently performed to rapidly evaluate the left eye.

## 2 Diagnosis: Retrobulbar Hematoma

The POCUS of the eye demonstrated an anechoic area posterior to the globe with posterior globe tenting consistent with a retrobulbar hematoma (RBH). (Fig 1 and 2) Intraocular pressure (IOP) was markedly elevated at 64 mmHg in the affected eye, compared with 20 mmHg in the unaffected. An emergent lateral canthotomy was prepared concurrently with CT, which confirmed a large left RBH (Fig 3). A lateral canthotomy with inferior cantholysis was performed, which reduced the IOP to 18 mmHg.

**Figure 1:**
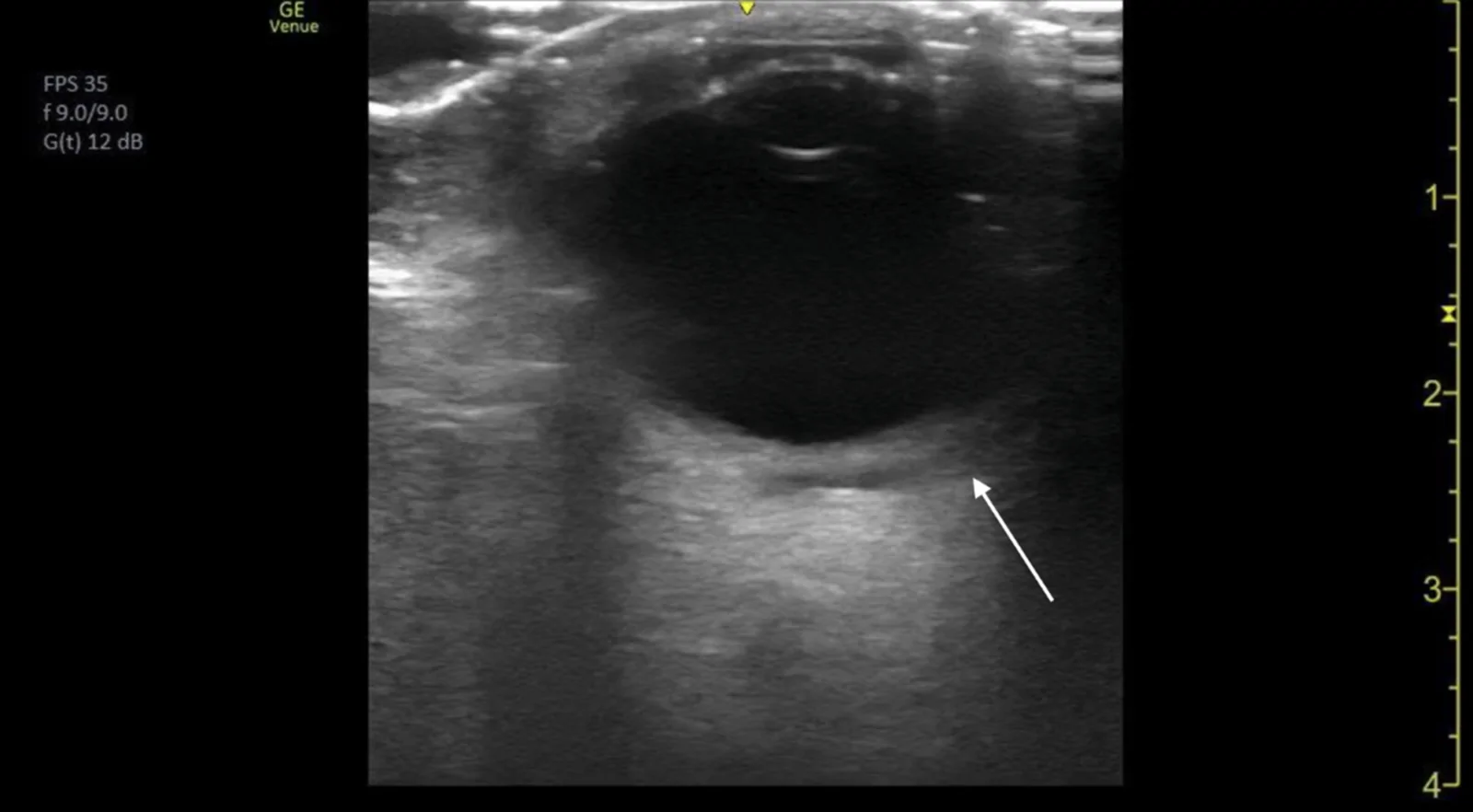
Point-of-care ultrasound, using a high frequency linear probe, of the eye showing an anechoic area posterior to the globe (arrow) consistent with a retrobulbar hematoma.

**Figure 2:**
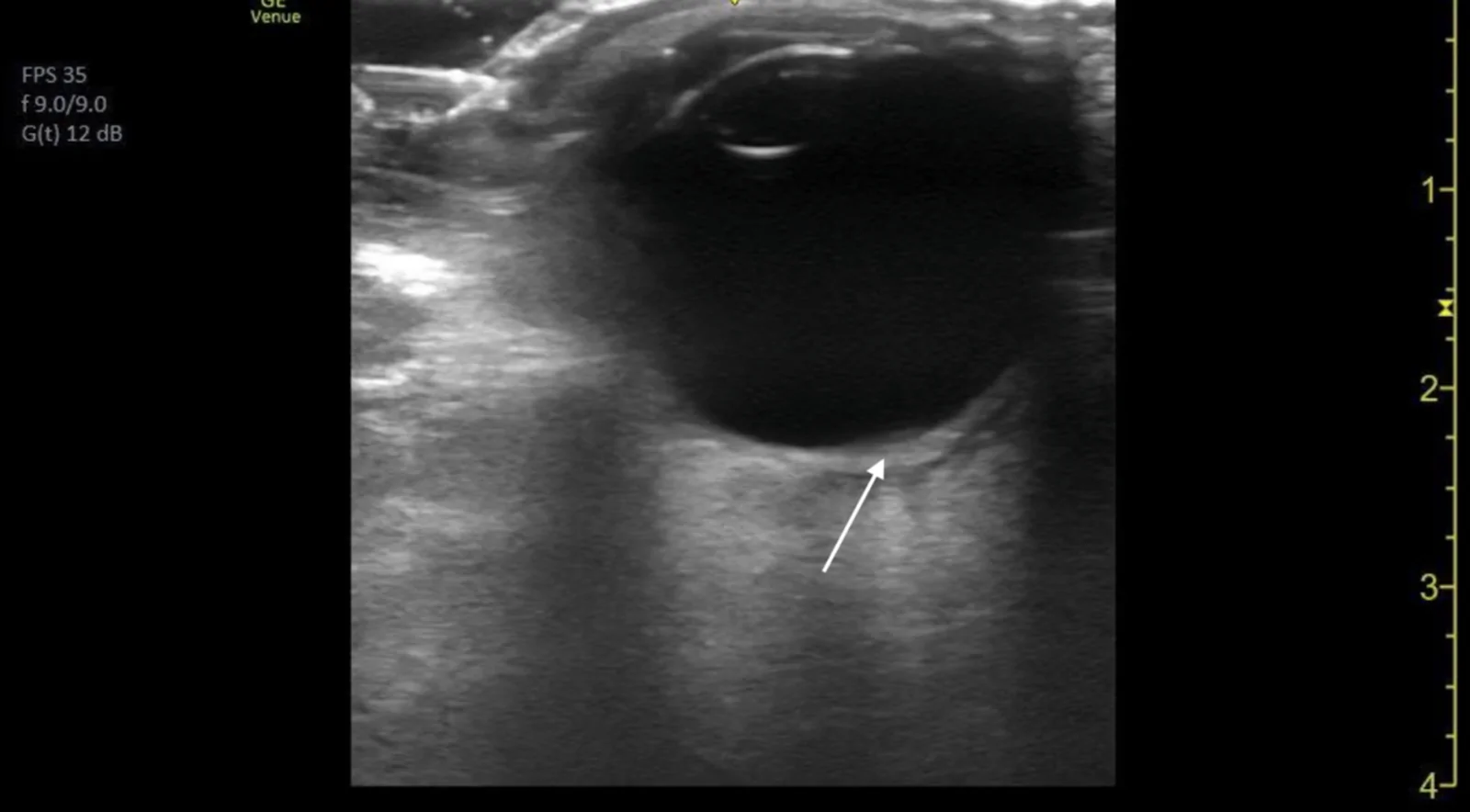
Point-of-care ultrasound of the eye demonstrating tenting of the posterior globe, “guitar pick” sign, (arrow), indicating increased ocular pressure.

**Figure 3:**
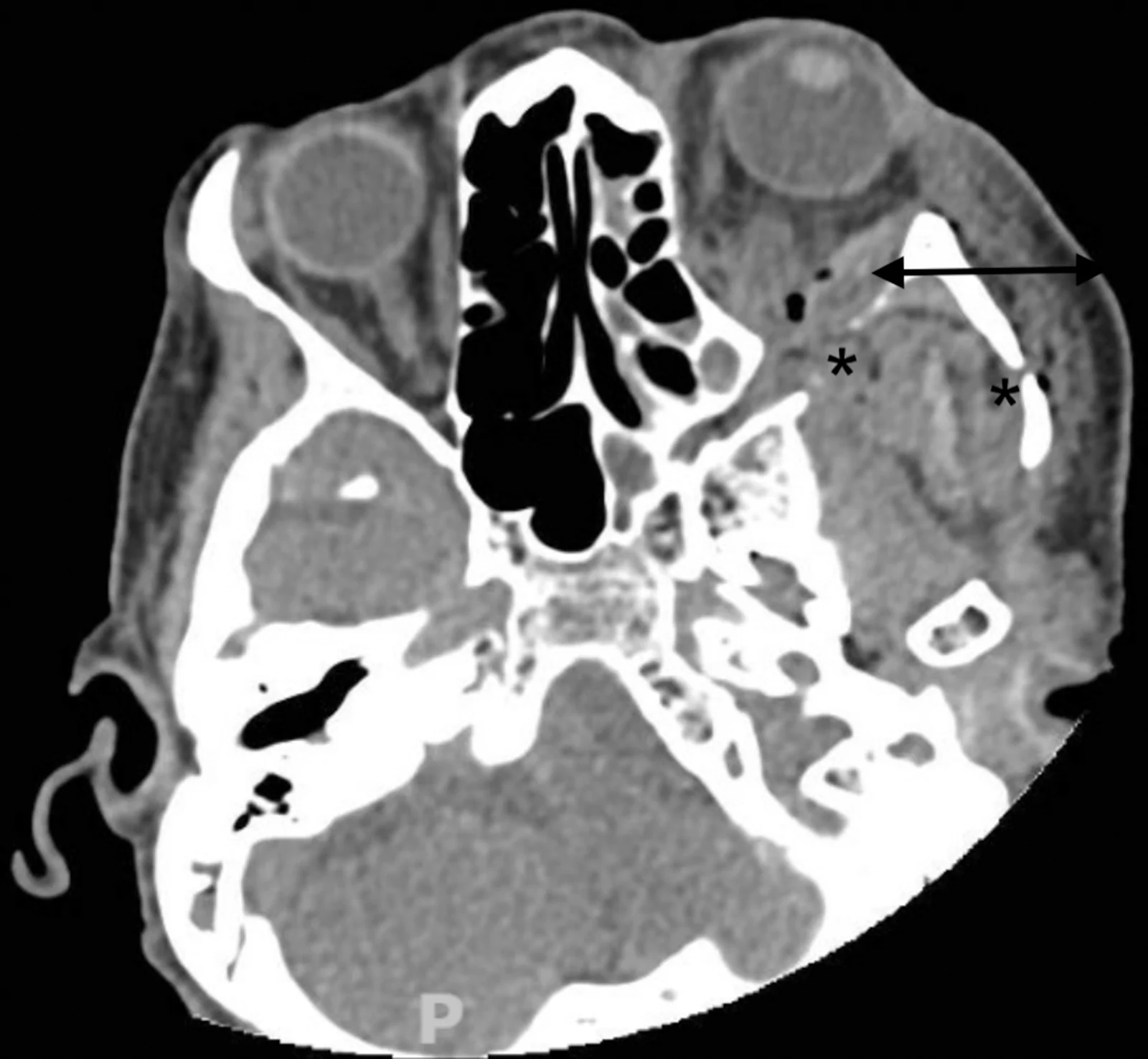
Axial noncontrast computed tomography demonstrating a hyperdense retrobulbar hematoma (arrow) measuring 1.5 x 0.7 cm within the lateral left intraconal space with associated proptosis, retro-orbital fat stranding, and intraconal air. Comminuted fractures of the left maxillary sinus walls, orbital floor, lamina papyracea, and lateral orbital wall are noted (arrowheads) with hemorrhagic opacification of the left maxillary sinus.

RBH is a sight-threatening emergency in which hemorrhage accumulates within the confined retrobulbar space, elevating intraorbital pressure and compromising perfusion to the optic nerve and retina. Irreversible vision loss can occur within 60 to 120 minutes without intervention.^1^ Diagnosis has traditionally relied on clinical findings including proptosis, relative afferent pupillary defect, elevated intraocular pressure, and diminished visual acuity and is often confirmed with CT.

Point-of-care ultrasound (POCUS) offers a rapid bedside alternative. Sonographic findings of RBH include a hyperechoic or heterogeneous collection posterior to the globe, and distortion of the posterior globe contour.^2^ Additional sonographic findings include the “guitar pick” sign which indicates increased intraorbital pressure and has been associated with the development of orbital compartment syndrome.^3^ Lateral canthotomy with inferior cantholysis is the definitive emergency department intervention, releasing the inferior crus of the lateral canthal tendon to decompress the orbit and restore vascular perfusion.^4^

## Funding and Support

By *JACEP Open* policy, all authors are required to disclose any and all commercial, financial, and other relationships in any way related to the subject of this article as per ICMJE conflict of interest guidelines (see www.icmje.org). The authors have stated that no such relationships exist.

## Ethics declaration

Written informed consent to take part in the study and to publish the article has been obtained from all participants or their legal representatives. The privacy rights of participants have been observed.

This study was performed in compliance with relevant laws, regulatory frameworks and guidelines where the research took place. Ethics committee approval was not required under relevant laws and institutional guidelines. not applicable, IRB approval is not necessary for case reports

This research follows the CARE guidelines and the CARE checklist.

## Conflict of Interest

All authors have affirmed they have no conflicts of interest to declare.
